# Optimization of the Pentachlorophenol Adsorption by Organo-Clays Based on Response Surface Methodology

**DOI:** 10.3390/ma15207169

**Published:** 2022-10-14

**Authors:** Soufiane El Mahmoudi, Abdellah Elmchaouri, Assya El kaimech, Antonio Gil

**Affiliations:** 1 INAMAT^2, Departamento de Ciencias, Universidad Pública de Navarra, Campus de Arrosadía, 31006 Pamplona, Spain; 2 Laboratory of Physical Chemistry & Bioorganic Chemistry, Faculty of Science and Techniques Mohammedia, University Hassan II of Casablanca, Mohammedia 20650, Morocco

**Keywords:** adsorption, montmorillonite, organo-clay, pentachlorophenol, response surface methodology

## Abstract

The aim of this study is to optimize the adsorption of pentachlorophenol (PCP) using an organo-clay under the response surface methodology. The adsorbent was selected from a montmorillonite exchanged by various cations, such as Fe^3+^, Al^3+^, Zn^2+^, Mg^2+^, Na^+^, and modified by bromide cetyltrimethylammonium (CTAB) as surfactant. The obtained organo-montmorillonite was characterized using several techniques, such as Fourier-transform infrared spectroscopy (FTIR), X-ray diffraction (XRD), thermogravimetric analysis (TGA), scanning electron microscopy (SEM), and nitrogen adsorption, performed at −196 °C. The results showed an increase in basal space from 1.65 to 1.88 nm and a decrease in the specific surface and pore volume, with an increase in pore diameter, including the presence of characteristic bands of -CH_2_- and -CH_3_- groups at 2926 and 2854 cm^−1^ in the FTIR spectrum after the modification. The optimization of PCP removal by clay adsorbents is achieved using the response surface methodology (RSM) with a four-factor central composite model, including pH of solution, mass of adsorbent, contact time, and initial concentration. The results proved the validity of the regression model, wherein the adsorption capacity reaches its maximum value of 38 mg/g at a lower adsorbent mass of 20 mg, pH of 6, contact time (t_c_) of 5 h, and initial concentration of 8 mg/L.

## 1. Introduction

Phenolic compounds, one type of priority pollutants in the aquatic environment, have attracted the attention of researchers as they have many harmful effects on human health even in low concentrations [1]. Pentachlorophenol (PCP) is one of the most commonly used chlorinated phenols, serving as a pesticide, herbicide, and preservative for wood and leather [2]. The environmental protection agency has classified chlorophenols, especially PCP, as priority pollutants due to their toxicity in the environment [3,4]. According to The Indiana Department of Environmental Management Bureau of Water—Water Quality Standards Section cancer criterion for human health, the acceptable amount of PCP in drinking water sources is 2.8 μg/L [5]. Therefore, it is essential to eliminate these toxic elements present in natural environments (soil and water) or to reduce their quantity below the admissible thresholds defined by the standards.

A promising technique to remove pesticides from an aqueous solution is adsorption on low-cost materials [6,7]. Adsorption is an effective wastewater treatment procedure applied by industries to decrease hazardous organic/inorganic contamination in effluents. Clay minerals have been employed as low-cost adsorbents for water cleaning and many efforts have been focused on heavy metals and dye [8,9,10], especially on montmorillonite for the adsorption of pesticides [11]. These adsorbents are considered efficacious and cost-effective, and their regeneration possibility after use is satisfactory. Natural clays are used after a previous purification step, which results in a material with advanced physico-chemical properties [12,13]. These materials, once in the water, form a suspended colloidal matter endowed with interesting sorption properties. Moreover, clay minerals are increasingly used as natural nanomaterials as they have no negative effects on the environment. The extensive use of clay is due to the octahedral (Al or Mg) or tetrahedral layers [14]. Nano-clay is used in different matrices to prepare nanocomposite materials and its addition leads to different enhanced properties of the obtained nanocomposite material. For example, the addition of nano-clay in the polymer matrix results in enhanced mechanical, diffusional barrier, fire retardant, and ultraviolet (UV) resistance properties of the material as well as thermal resistance [15,16].

Clays have an hydrophilic surface in their natural state [17]. The modification by cationic surfactants improves the surface hydrophobic character and leads to surface charge reversal from negative to positive [18,19]. Organophilic clay can be obtained by a simple cation exchange, which serves to fix the molecules of surfactant in the interlayer space [20]. The organically modified montmorillonite synthesized with the surfactant had a larger interlayer space and higher values of wetting contact angles, which could have a potential for adsorption of organic contaminant [21].

Response surface methodology is a technique used to optimize various processes by quantifying the relationship between one or more measured responses and the vital input factor [22]. RSM is an appropriate approach and widely used not only for studying PCP, but also for developing and optimizing a wide range of engineering systems in several industry processes [23,24]. RSM was effectively used in the pharmaceutical industry [25] for the optimization and modeling of operating parameters of a wide variety of microbial products [26]. In addition, it was applied for the modeling and optimization of operating parameters for water desalination [27] as well as other applications in food processes, such as extraction, drying, blanching, enzymatic, hydrolysis and clarification, production of microbial metabolites, and formulation [28,29,30].

In this work, we study the main effects that impact PCB adsorption on a montmorillonite modified with bromide cetyltrimethylammonium (CTAB) surfactant using statistical tools. The traditional method remains limited since it does not consider all of the possible combinations; however, this is possible using a statistical design, such as response surface methodology (RSM) [31,32]. RSM employs a group of mathematical and statistical techniques based on the fit of empirical models with experimental data obtained as part of the experimental design [33]. To achieve this objective, linear or square polynomial functions are used to describe the system studied and, consequently, to explore the experimental conditions until optimization is reached.

## 2. Experimental Procedure

### 2.1. Materials and Reagents

Throughout this study, montmorillonite K-10 was purchased from Sigma-Aldrich (Burlington, MA, USA) and used as the starting clay (as received, in the form of powder). Bromide cetyltrimethylammonium (CTAB, purity ≥ 99.0%) was purchased from Sigma Aldrich (Burlington, MA, USA). Pentachlorophenol (PCP), which was used as an adsorbate, was obtained from Fluka (Buchs, Switzerland). Moreover, FeCl_3_·6H_2_O (98 %) was purchased from SDFCL (Chennai, India), AlCl_3_ and MgCl_2_ were purchased from Sigma-Aldrich, NaCl (≥ 99.0%) from Fluka (Buchs, Switzerland), and ZnCl_2_ from SCP science (Montreal, Canada).

### 2.2. Preparation of the Adsorbents

The preparation of the various organophilic montmorillonites was carried out according to two stages. The first step employs a cation exchanger, and the second step involves obtaining a clay that presents only one possible exchangeable cation. The cations used in this study are Mg^2+^, Na^+^, Al^3+^, Fe^3+^, and Zn^2+^. The experiments were performed according to the method detailed in [34]. Briefly, 5 g of montmorillonite was slowly added to 100 mL of an aqueous solution of cations and the resulting mixture was stirred for 4 h. Then, the suspension was centrifuged, and the operation in principle was carried out four times to optimize the exchange. Thereafter, the clay was rinsed several times to remove the excess of salt, and chloride ions were removed from the washing solution using silver nitrate. Next, the clay was dried in an oven at 80 °C. The modification by bromide cetyltrimethylammonium was carried out to prepare a suspension of 5 wt% of Mt-cation in distillated water, with 0.8 g of CTAB added to the solution and stirred for 2 h at 60 °C. The resulting slurry was centrifuged and washed with distillated water until its disappearance in the foam, then dried at 70 °C for 16 h. The solid obtained was denoted as Mt-Cation-CTAB.

### 2.3. Preliminary adsorption test

To select the best adsorbent material, a preliminary study was carried out in two steps. The first step involves the adsorption of PCP on various montmorillonites synthesized under classical conditions and the second step is a comparison of their adsorption capacities toward PCP. To carry out the adsorption tests, 1 g of each montmorillonite sample (Mt, Mt-CTAB, Mt-Na-CTAB, Mt-Mg-CTAB, Mt-Fe-CTAB, Mt-Al-CTAB, and Mt-Zn-CTAB) was introduced into a series of 250 mL Erlenmeyer flasks and 100 mL of PCP with a concentration of 10 mg/L. These flasks were placed under magnetic stirring at a speed of 400 rpm for 12 h at room temperature. The adsorption capacity was calculated at an equilibrium time of 12 h using the following Equation (1):

(1) $$Q_{e}=\frac{V\left(C_{\mathrm{int}}-C_{e}\right)}{m}$$

where Q_e_ (mg/g) is the amount of PCP adsorbed at equilibrium time, C_int_ and C_e_ (mg/L) are the initial and equilibrium concentrations of PCP in the solution phase, respectively, V (L) is the solution volume, and m (g) is the mass of adsorbent.

The concentration of the PCP was evaluated by JASCO-750 UV (Tokyo, Japan) spectrophotometer at a wavelength of 320 nm [35,36]. The adsorption capacity of each adsorbent material is summarized in Table 1. From these results, Mt-Fe-CTAB was selected for further optimization studies by RSM.

## 3. Materials Characterization

### 3.1. Infrared Analysis

**Figure 1:**
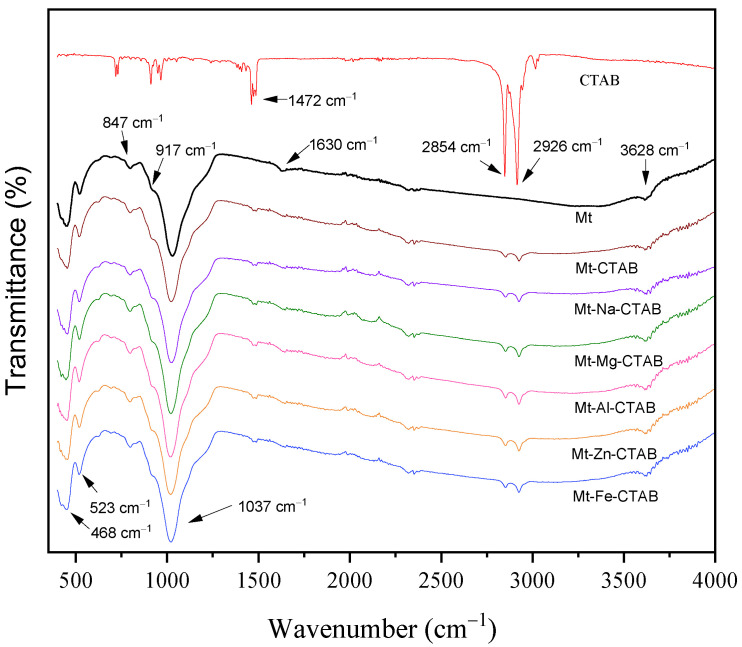
FTIR spectra of CTAB and CTAB-modified Mt.

The FTIR analysis was recorded at room temperature in the mid-IR range (400–4000 cm^−1^) using a Spectrum Two FT-IR Spectrometer apparatus from Perkin Elmer (Waltham, MA, USA) equipped with ATR accessory with a single reflection diamond crystal. In addition, the spectra were obtained by collecting four scans using a 4 cm^−1^ resolution. The FTIR spectra of raw Mt, Mt-CTAB, Mt-Fe-CTAB, Mt-Al-CTAB, Mt-Zn-CTAB, Mt-Na-CTAB, and Mt-Mg-CTAB (see Figure 1) have almost the same appearance. All the FTIR spectra have a band at 3628 cm^−1^, which is attributed to the elongation vibration of the OH groups of the octahedral layer of montmorillonite. Other bands observed at 917 and 847 cm^−1^ correspond to the bending vibrations of AlOH and MgOH [37]. A very intense band at 1037 cm^−1^ corresponds to the valence vibrations of the Si-O bond of tetrahedral sheets. The absorption bands at 523 and 468 cm^−1^ belong to the bending vibrations of the Si-O-Al and Si-O-Si bonds. Another band centered around 1630 cm^−1^ is related to the deformation vibrations of H_2_O molecules adsorbed between the sheets.

To examine the functional groups of each material studied, the FTIR spectra of montmorillonites without CTAB- and CTAB-modified montmorillonites were compared. A few new bands appeared in the spectra of the CTAB-intercalated MMT showing the existence of the characteristic functional groups of the surfactant. The first band at 1472 cm^−1^ corresponds to the shear vibrations of -CH_2_ and -CH_3_. Another band at 2926 and 2854 cm^−1^ corresponds to asymmetric and symmetric stretching vibrations of -CH_2_ and -CH_3_. In addition, a small decrease in the free OH bands of the water molecules and the OH bands at 3450 and 3600 cm^−1^ was observed, confirming the decrease in the amount of water caused by the hydrophobic nature of the organo-montmorillonite. These results suggest that CTAB was well adsorbed on the surface of the montmorillonite.

### 3.2. Thermogravimetric Analysis

**Figure 2:**
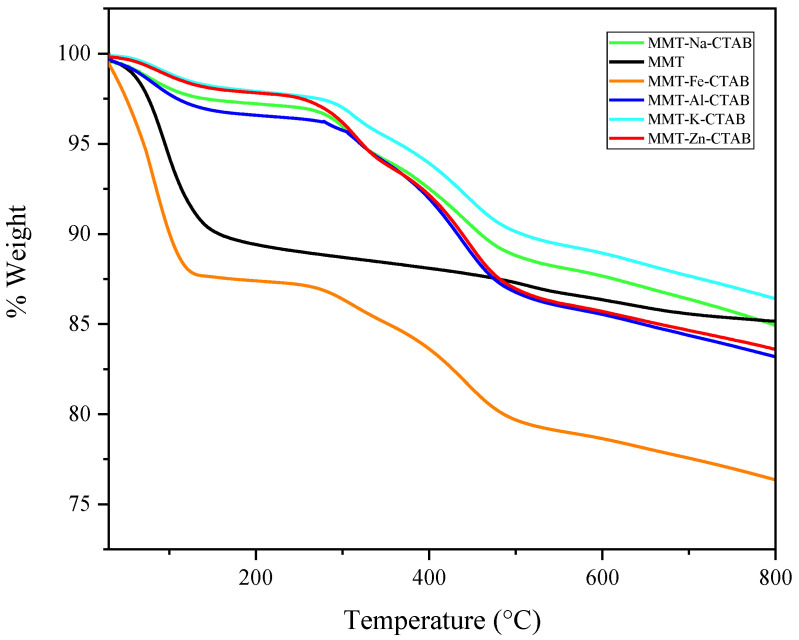
Thermogravimetric analysis of raw Mt, Mt-Fe-CTAB, Mt-Al-CTAB, Mt-Mg-CTAB, Mt-Na-CTAB, and Mt-Zn-CTAB.

The thermogravimetric analysis was performed with a PerkinElmer TGA 4000 (Waltham, MA, USA) in a flowing nitrogen atmosphere (20 mL/min) at a heating rate of 10 °C/min from 30 to 800 °C. The TGA curves of all the CTAB-modified montmorillonites (see Figure 2) have overall similar shapes with a difference in the percentage of CTAB loss. The CTAB-modified montmorillonites showed a moderately low plateau from 50 to 200 °C compared with the raw Mt, indicating that the CTAB-modified Mt had less free water than the raw Mt. Therefore, the adsorbed CTAB reduced the interfacial free energy of the Mt, indicating the formation of a hydrophobic surface, which was consistent with the IR results. Two clearly visible mass losses on the TGA curves of the CTAB-modified Mt at 289 and 467 °C were due to the pyrolysis decomposition of intercalated and adsorbed CTAB between the layers. In addition, two other mass losses at 603 and 649 °C are attributed to the dehydroxylation of montmorillonite sheets.

### 3.3. Scanning Electron Microscopy Analysis

**Figure 3:**
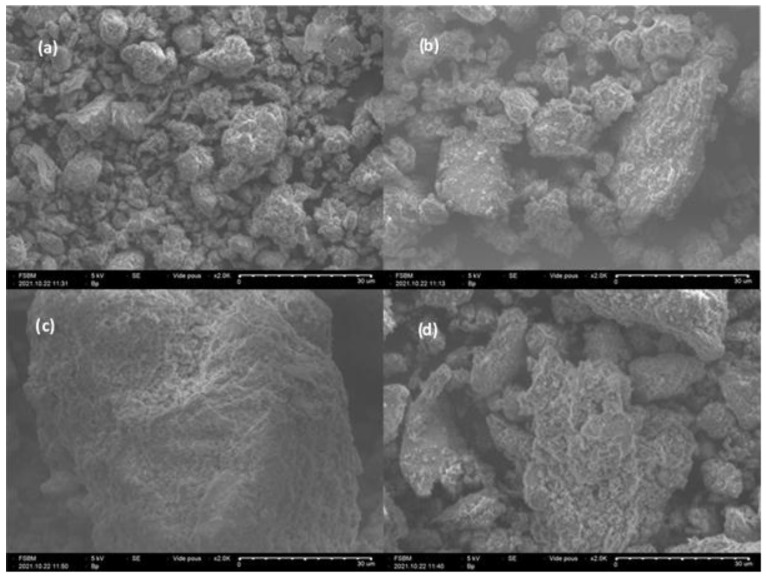
SEM micrographs of (**a**) Mt, (**b**) Mt-CTAB, (**c**) Mt-Fe, and (**d**) Mt-Fe-CTAB.

Scanning electron microscopy analysis for the raw Mt (see Figure 3a) shows a dense, agglomerated, and smooth structure, while in the Mt-CTAB (Figure 3b), upon addition of CTAB, a less dense structure was obtained with a rough surface. Similar to the fact that the cation exchange by iron ions (Figure 3c) leads to a slightly rough surface, the addition of CTAB leads to a surface rough with the formation of small aggregates compared with Mt-CTAB and Mt-Fe, which explains their high adsorption capacity. According to the results, we can conclude that the modification of Mt by the CTAB surfactant leads to a rough surface with the formation of small size aggregates. Moreover, the ion exchange increased the surface area of Mt, according to our previous laboratory study [38]. Furthermore, the SEM analysis confirms that the modification by CTAB was well conducted.

### 3.4. Point of Zero Charge (pHpzc)

**Figure 4:**
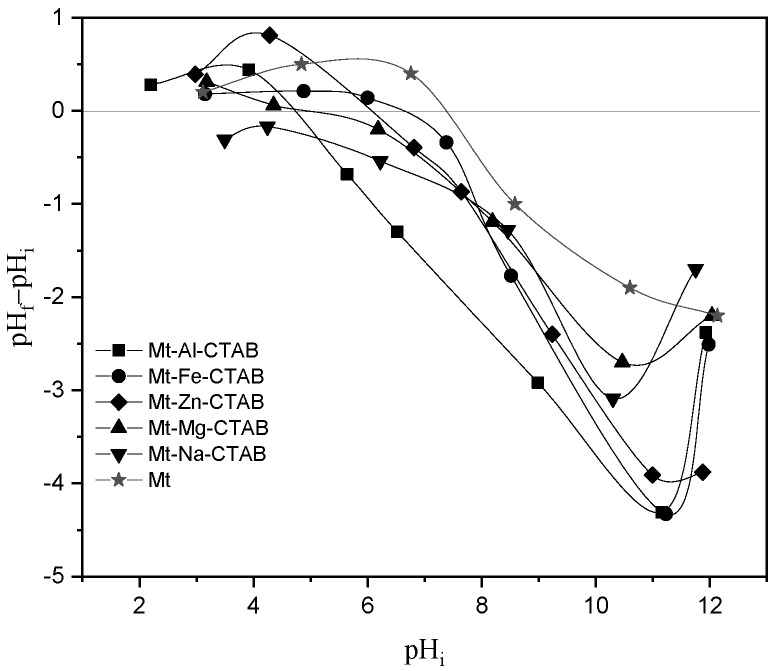
The pHpzc of Mt and modified organo-clays.

The pHpzc or pH of the point of zero or null charge corresponds to the pH value, in which the net charge of the surface of the adsorbents is null. This parameter is very important in adsorption phenomena, especially when electrostatic forces are involved in the mechanisms. The pHpzc values of Mt and organo-Mt are determined in the method described by Benhouria et al. [39]. In each Erlenmeyer flask, 50 mg of adsorbent is added to 50 mL of distilled water for a pH range from 2 to 12. The pH of each solution was adjusted by the addition of HCl and/or NaOH and stirring for 24 h. Then, the final pH was measured. The results of the isoelectric point of organo-Mt and raw Mt are presented as ΔpH = (pH_f_-pH_i_) as a function of pH_i_ (see Figure 4). The pHpzc values are included between 3.89 and 5.97 for the modified Mt and 6.5 for the raw Mt. Therefore, the modification of raw Mt by the CTAB surfactant changes the surface charge from negative to positive when the pH is lower than 6.5.

### 3.5. Nitrogen Adsorption and Textural Analysis

**Figure 5:**
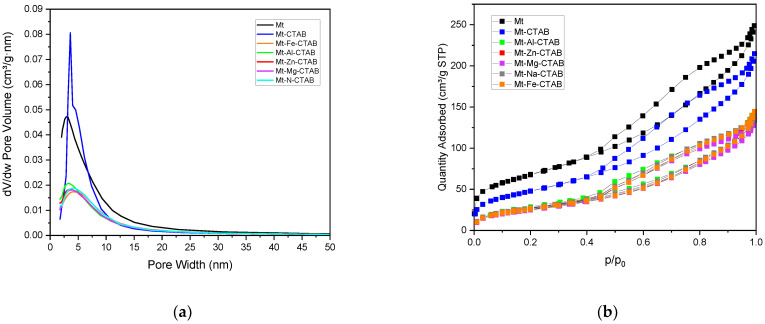
The pore size distribution estimated by the BJH model (**a**) of Mt- and CTAB-modified Mt and N_2_ adsorption-desorption isotherms (**b**).

To study the textural properties of the prepared Mt-M-CTAB, the N_2_ adsorption capacity was measured at −196 °C using a Micromeritics ASAP 2020 Plus model (Norcross, GA, USA). The isotherm plot (see Figure 5) represents a type II adsorption behavior, according to the UPAC classification [40,41]. After desorption of the adsorbed N_2_, a type-H3 hysteresis loop characteristic of a mesoporous structure of the organo-clay was obtained (see the distribution of the pore size obtained from the BJH model, Figure 5). This type of hysteresis loop characterizes the materials that consist of aggregates generating pores of non-uniform size. The quantitative results of the textural analysis are summarized in Table 1. As can be seen, the specific surface area and the pore volume of the organo-clay decrease after the modification of the clay by CTAB, indicating the occupation of the surface by the CTAB molecules. Upon the cation exchange with CTAB, the gallery space available for adsorption was significantly reduced. In addition, the bulky size of CTAB occupied more potential sorption sites in the inter-gallery space, leading to an increase in pore diameter [42].

### 3.6. XRD Analysis

**Figure 6:**
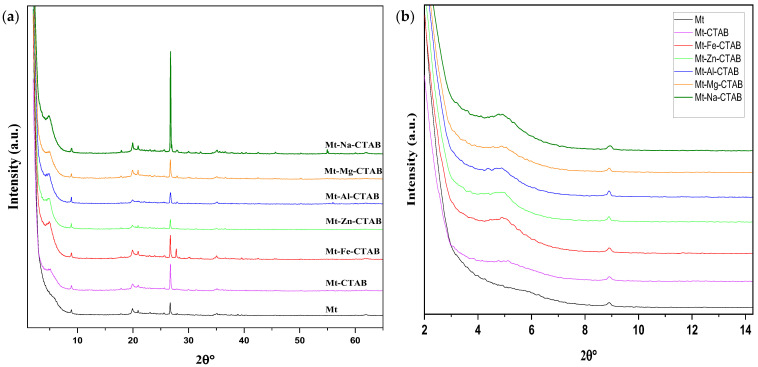
XRD patterns of the Mt- and CTAB-modified Mt. (**a**) range between 2 and 65°; (**b**) low range between 2 and 14°.

The X-ray characterization was carried out using a Siemens D 5000 model (Plano, TX, USA) diffractometer equipped with an Ni-filtered Cu-Kα radiation source (λ = 0.1548 nm). The X-ray diffractogram of the Mt exhibited a low intensity reflection peak at 2θ = 5.34° (see Figure 6), according to the results described by other authors [43,44]. As seen in Figure 1, Mt possesses a number of sharp peaks that correspond to various impurities: Quartz between 19.8 and 35° and Feldespat between 26.8 and 27.8° [45]. The high intensity basal reflections indicate a large number of repetitive clay platelets, which is visible for organo-clays. However, the raw Mt used had a low degree of laminar stacking, which explains the low basal reflection intensity. The basal space increased after the modification from 1.65 to 1.88 nm, which may explain the successful implementation of intercalation [46].

### 3.7. Batch Adsorption Experiments

An adequate choice of the parameter variation domains is an essential condition to establish an accurate model that perfectly describes the study process. Before organizing the adsorption tests that will allow us to answer the remaining questions, it is imperative to define the study domain of each of the factors. This choice was made based on literature and preliminary tests. As indicated in the majority of literature reports, the solubility of PCP in water ranges between 10 and 20 mg/L. Since PCP is a weak acid (pKa = 4.35), its solubility increases significantly with the increasing pH [47]. Therefore, we selected the experimental study region with 40 spans, a pH range from 5 to 9, a contact time range from 1 to 13 h, an adsorbate mass from 10 to 50 mg, and an adsorbent concentration from 2 to 10 mg/L. According to our study, all of the PCP solutions were prepared with deionized water.

## 4. Statistical Analysis

Optimization of PCP removal by clay adsorbents is achieved using RSM with a central composite model. A rotatable central composite design uniform with rotational isovariance and uniform accuracy was used to investigate the empirical relationships between two responses (PCP removal efficiency and adsorbed amount). This matrix has many advantages, particularly high resolution and minimal number of trials [33]. For four factors (pH of solution, mass of adsorbent, contact time, and initial concentration), a full five-level factorial design requires 54—625 experiments, while the centered composite design with rotational isovariance requires only 31 experiments. The number of experiments for this design is obtained from the following equation [22]:

(2) $$N=k2+2k+C_{p}\;$$

where *k* represents the number of factors, and *C_p_* represents the number of replicates of the central point.

Design expert statistical software (JMP 2013, v.13.0.0, Brie Comte Robert, France) was used for model fitting as well as the significance for adsorption efficiency and adsorbed quantity of PCP. The four factors were evaluated at five levels (−α, −1, 0, +1, +α) and the coded values were calculated according to Equation (3), as shown in Table 2.

(3) $$X_{i}=\frac{x_{i}-x_{0}}{\Delta x}$$

where *X_i_* is the dimensionless value of an independent variable, *x_i_* is the real value of an independent variable, *x*_0_ is the value of *X_i_* at the center point, and *Δx* is the step range.

Each response can be expressed as a quadratic equation as follows [48,49]:

(4) $$Y=\beta_{0}+\sum_{i=1}^{k}\beta_{i}X_{i}+\sum_{i=1}^{k}\beta_{ii}X_{i}^{2}+\sum_{i=1}^{k}\sum_{j=1}^{k}\beta_{ij}X_{i}X_{j}+\varepsilon$$

where *Y* denotes the response, *β*_0_ denotes the constant coefficient, *X_i_* and *X_j_* represent the coded values for independent variables, and *βij*, *βii*, *βi*, *k*, and *ε* denote the interaction, quadratic, linear coefficients, the number of factors studied and optimized in the experiment, and the random error, respectively [50].

The resulting model in terms of coded values versus response is summarized in the following equation [51]:

(5) $$\begin{array}{cc} Y=\beta_{0}+\beta_{1}{Χ}_{1}+ & \beta_{2}{Χ}_{2}+\beta_{3}{Χ}_{3}+\beta_{4}{Χ}_{4}+\beta_{11}{Χ}_{12}+\beta_{22}{Χ}_{22}+\beta_{33}{Χ}_{32}+\beta_{44}{Χ}_{42}\\ & +\beta_{12}{Χ}_{1}{Χ}_{2}+\beta_{13}{Χ}_{1}{Χ}_{3}+\beta_{23}{Χ}_{2}{Χ}_{3}+\beta_{14}{Χ}_{1}{Χ}_{4}+\beta_{24}{Χ}_{2}{Χ}_{4}\\ & +\beta_{34}{Χ}_{3}{Χ}_{4}+\varepsilon \; \end{array}$$

The first part (experiments 1 to 16, see Table 3) is the factorial part, which constitutes the eight vertices of the unit cube. The second part (experiments 17 to 24) is the axial part, which constitutes the points on the axes of the reference frame used. Each of these experiments is used for one of the factor levels outside of the usual operating values. Finally, the last part of the composite design (experiments 24 to 31) is constituted by a repetition (*C_p_* = 7 times) of the experiment that is qualified as central (with all the factors fixed at their average level). In a statistical context, it is interesting to repeat some experiments several times since the random nature of the phenomenon will lead to the fact that the observed responses will not be equal.

The effects of all the factors studied along with the statistical values of t-student and the observed probability (*p*-value) are grouped in the coefficient effects (see Table 4). The t-student values are used to determine the significance of the coefficients for each parameter. In general, the larger the magnitude of t, the smaller the *p*-value, and the more significant the corresponding coefficient term. From these results, the following Equation (6) can be proposed:

(6) $$Q_{e}=19.106754-1.873911pH-6.20915m+5.1904503C_{\mathrm{int}}-1.708197C_{{\mathrm{int}}^{2}}\;$$

According to the results included in Table 4, the second-order response surface model fitting in the form of the analysis of variance (ANOVA) is summarized in Table 5. The significance of the regression model was evaluated using the Fisher distribution. A larger F-value indicates a better fit of model to the experimental extraction efficiency. In addition, the null-hypothesis test (*p*-value) is considered, where a *p*-value less than 0.05 indicates the design variable of a model contributing less than 5% change in the response. Therefore, the variable with a larger F-value and *p* < 0.05 was considered significant.

**Figure 7:**
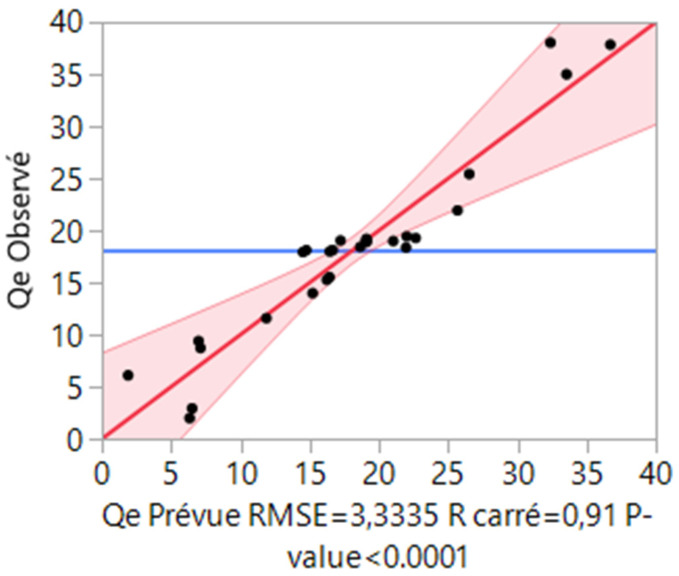
Relation between actual and predicted adsorbed amount of PCP on Mt-Fe-CTAB.

**Figure 8:**
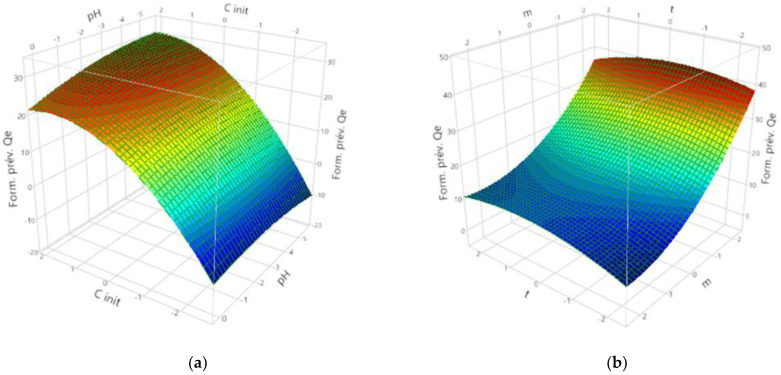
Representation of the response surface in three-dimensional space. (**a**) Interactive effect of initial concentration and pH. (**b**) Interactive effect of contact time and mass of adsorbent.

The relation between the actual and predicted adsorbed amount of PCP was included in Figure 7. The results show evidence of the validity of the regression model. Finally, as shown in Figure 8a, it was clear that the adsorption capacity increased gradually with the increase in pH, and the optimal adsorption capacity was observed at a pH of 6. This result can be explained by the electrostatic interaction between the negatively charged PCP (the PCP is a weak acid and its pKa is 4.7, which indicates that at pH > 4.7, it deprotonates as negatively charged species PCP [52]) and the positively charged surface of the modified clay. On the other hand, the initial concentration proved to have a significant effect on the adsorption capacity. As can be seen in Figure 8a, the adsorption capacity increases with the increase in the initial concentration. This increase may be due to the increase in the mass transfer driving force upon increasing the initial PCP concentration, which leads to the increase in the diffusion of PCP molecules in the solution to the surface of Mt-Fe-CTAB. In addition, increasing the initial PCP concentration increases the probability of collision between the PCP molecules and the adsorption sites of the Mt-Fe-CTAB. The effect of the mass of adsorbent present in Figure 8b shows that the highest adsorption capacity was reached when the mass is lower than 20 g. The PCP removal rate is affected by the mass of adsorbent, which is due to the fact that when the mass of the Mt-Fe-CTAB decreases, the crystallite tends to disperse, which leads to an increase in the total surface area of adsorbent particles available for PCP fixation. This behavior can be explained by the fact that at higher adsorbent mass, the available number of PCP molecules in the solution is not sufficient to combine completely with all the effective adsorption sites on the Mt-Fe-CTAB, resulting in a state of surface equilibrium, and consequently in a decrease in the adsorption capacity of PCP.

The predicted optimal conditions based on the RSM were: An initial pH of 6, a mass of adsorbent of 20 g, an initial concentration of 8 mg/L, and a contact time of 5 h. The maximum adsorption capacity of PCP was 33.7 ± 4.54 mg/g. Confirmatory experiments were conducted with the parameters to check the accuracy of the optimum set of parameters and the adsorption capacity was found to be 38 mg/g. The experimental value was close to the results obtained from RSM, which validated the findings of response surface optimization.

## 5. Conclusions

In this work, the preparation of organo-clay from a montmorillonite modified with CTAB as a surfactant through intercalation processes was confirmed by the performed analyses. FTIR analysis confirmed the presence of CTAB in Mt using the existing characteristic functional groups of CTAB. XRD analysis was successfully carried out to prove the intercalation of CTAB in the interlayer space. Herein, the analysis resulted in an increase in basal space from 1.65 to 1.88 nm, which confirms that the intercalation was carried out and lead to an increase in basal space. Moreover, TGA analysis confirmed the modification of Mt by CTAB, through the loss of mass at 289 and 467 °C due to the pyrolysis decomposition of intercalated and adsorbed CTAB. Furthermore, SEM analysis presented the surface change after intercalation from a dense, agglomerated, and smooth structure to a rough surface with the formation of small aggregates. This modification resulted in a decrease in the specific surface and pore volume as well as an increase in the pore diameter. In conclusion, the metal ion led to an increase in the pH range of the positive charge of intercalated Mt.

The optimal condition for high adsorption capacity was obtained with a minimum number of experiments using the RSM central composite model, for four factors: Solution pH, adsorbent mass, contact time, and initial concentration. The results proved the validity of the regression model, wherein the adsorption capacity reached its maximum value of 38 mg/g at a lower adsorbent mass of 20 g, pH of 6, initial concentration of 8 mg/L, and contact time of 5 h. 

## Figures and Tables

**Table 1:** Adsorption capacity of the adsorbent materials after 12 h of contact, C_0_ = 10 mg/L, and textural properties of the adsorbents.

Adsorbent	PCP, Amount Adsorbed (mg/g)	S_BET_ (m^2^/g)	S_ext_ (m^2^/g)	d_p_ (nm)	V_p_ (cm^3^/g)
Mt	13.50	241	130	6.22	0.375
Mt-CTA	21.28	173	123	7.35	0.319
Mt-Fe-CTA	34.57	96	94	8.86	0.213
Mt-Al-CTA	26.15	105	84	8.06	0.213
Mt-Zn-CTA	28.52	98	86	8.25	0.203
Mt-Mg-CTA	24.59	92	82	8.61	0.197
Mt-Na-CTA	26.82	100	84	8.28	0.208

**Table 2:** Experimental areas of the factors studied in the optimization of PCP adsorption capacity on Mt-Fe-CTAB.

Variables (*X_i_*)	−2	−1	0	+1	+2	Δx
X_1_ = pH	5	6	7	8	9	1
X_2_ = t_c_ (h)	1	4	7	10	13	3
X_3_ = m (mg)	10	20	30	40	50	10
X_4_ = C_int_ (mg/L)	2	4	6	8	10	2

X_1_ = (x_1_ − 7)/1; X_2_ = (x_2_ − 7)/3; X_3_ = (x_3_ − 30)/10; and X_4_ = (x_4_ − 6)/2.

**Table 3:** Experimental design of the optimization of the PCP adsorption capacity on the Mt-Fe-CATB with the responses recorded for each trial.

Order	pH	t_c_	m	C_int_	pH	t_c_	m	C_int_	Q_e_
1	−1	1	−1	1	(6)	(10)	(20)	(8)	35.0000
2	1	−1	−1	−1	(8)	(4)	(20)	(4)	18.0000
3	0	0	0	0	(7)	(7)	(30)	(6)	19.0850
4	−1	−1	−1	−1	(6)	(4)	(20)	(4)	18.3660
5	1	1	1	−1	(8)	(10)	(40)	(4)	1.9935
6	0	0	0	2	(7)	(7)	(30)	(10)	19.2810
7	0	−2	0	0	(7)	(1)	(30)	(6)	15.2723
8	−1	−1	1	1	(6)	(4)	(40)	(8)	15.5229
9	0	0	0	0	(7)	(7)	(30)	(6)	18.9107
10	0	0	0	−2	(7	(7)	(30)	(2)	6.1002
11	0	0	0	0	(7)	(7)	(30)	(6)	19.0414
12	−1	−1	−1	1	(6)	(4)	(20)	(8)	38.0392
13	0	0	−2	0	(7)	(7)	(10)	(6)	37.8431
14	0	2	0	0	(7)	(13)	(30)	(6)	19.0414
15	−1	1	1	1	(6)	(10)	(40)	(8)	18.4314
16	1	−1	−1	1	(8)	(4)	(20)	(8)	21.9281
17	1	1	1	1	(8)	(10)	(40)	(8)	18.1209
18	2	0	0	0	(9)	(7)	(30)	(6)	17.9303
19	0	0	0	0	(7)	(7)	(30)	(6)	19.1939
20	0	0	2	0	(7)	(7)	(50)	(6)	11.5817
21	−1	1	1	−1	(6)	(10)	(40)	(4)	8.7255
22	0	0	0	0	(7)	(7)	(30)	(6)	19.2157
23	−2	0	0	0	(5)	(7)	(30)	(6)	19.4336
24	1	−1	1	−1	(8)	(4)	(40)	(4)	2.9248
25	0	0	0	0	(7)	(7)	(30)	(6)	19.0632
26	1	1	−1	−1	(8)	(10)	(20)	(4)	13.9869
27	−1	1	−1	−1	(6)	(10)	(20)	(4)	18.9869
28	1	1	−1	1	(8)	(10)	(20)	(8)	25.4248
29	1	−1	1	1	(8)	(4)	(40)	(8)	18.1209
30	−1	−1	1	−1	(6)	(4)	(40)	(4)	9.3954
31	0	0	0	0	(7)	(7)	(30)	(6)	19.2375

Q_e_: Equilibrium adsorption.

**Table 4:** Effects of model coefficients relating response to factors.

Term	Coefficient	Estimation	Standard Error	*t* Ratio	prob. > |t|
Constant	*β*_0_	19.106754	1.279135	14.94	<0.0001*
pH	*β*_1_	−1.812364	0.690812	−2.62	0.0184 *
T	*β*_2_	0.1847313	0.690812	0.27	0.7926
m	*β*_3_	−6.270697	0.690812	−9.08	<0.0001 *
C_init_	*β*_4_	5.1289034	0.690812	7.42	<0.0001 *
pH × t_c_	*β*_12_	−0.171569	0.846068	−0.2	0.8419
pH × m	*β*_13_	1.1662582	0.846068	1.38	0.187
t × m	*β*_23_	0.3574346	0.846068	0.42	0.6783
pH × C_init_	*β*_14_	−0.394199	0.846068	−0.47	0.6476
t × C_init_	*β*_24_	0.6147876	0.846068	0.73	0.4779
m × C_init_	*β*_34_	−0.151144	0.846068	−0.18	0.8605
pH × pH	*β*_11_	−0.194989	0.63287	−0.31	0.762
t_c_ × t_c_	*β*_22_	−0.576253	0.63287	−0.91	0.3761
m × m	*β*_33_	1.3126362	0.63287	2.07	0.0546
C_init_ × C_init_	*β*_44_	−1.69281	0.63287	−2.67	0.0166 *

**Table 5:** Estimated regression coefficients and corresponding F- and p-values for adsorbed PCP.

Source	Degree of Freedom	Sum of Squares	Mean Square	F-Value	*p*-Value
Model	14	1843.1399	131.6530	11.4947	<0.0001
Residual	16	183.2532	11.4530		
Total	30	2026.3931			

## Data Availability

All the data are available within the manuscript.
